# Methodological Evaluation of a P2C-Based ReMOT CRISPR/Cas9 System in *Aedes aegypti*

**DOI:** 10.3390/insects17050451

**Published:** 2026-04-24

**Authors:** Xiaohui Liu, Wenhao Wang, Xiaoxue Xie, Haotian Yu, Chunxiao Li

**Affiliations:** State Key Laboratory of Pathogen and Biosecurity, Beijing 100071, China

**Keywords:** gene editing, *Aedes aegypti*, ReMOT, CRISPR/Cas9

## Abstract

Mosquito-borne infectious diseases remain a major challenge to public health, highlighting the need for efficient and accessible gene editing approaches. Receptor-mediated ovary transduction of cargo (ReMOT) offers an alternative to embryonic microinjection, in which P2C, an ovary-targeting peptide, enables ovarian delivery of the editing components. However, key design parameters and operational boundaries of the P2C-based ReMOT system have not been clearly defined. Here, we performed a methodological evaluation of the P2C-mediated ReMOT CRISPR/Cas9 system in *Aedes aegypti*. Cas9-P2C fusion proteins with different configurations were constructed and assessed through ovarian targeting assays, in vitro cleavage analyses, and in vivo gene editing experiments. Our results show that full-length Cas9-P2C fusion proteins exhibit nuclease activity and enable effective ovarian delivery. In contrast, linear truncation of the P2C peptide markedly reduced ovarian targeting, indicating a dependence on structural integrity. Using this delivery strategy, we generated *kynurenine monooxygenase* (*KMO*) edited mosquitoes, demonstrating feasibility under the conditions tested. In addition, protein injection was also associated with reduced reproductive performance, providing physiological reference for ReMOT applications. Overall, this study defines the key design parameters and operational boundaries of the P2C-based ReMOT system, providing methodological guidance for its application and optimization in future mosquito genetic studies.

## Simple Summary

Traditional mosquito gene editing techniques rely on embryo microinjection. However, this method is technically challenging, requires expensive equipment, and has low success rates. An alternative strategy involves delivering gene editing proteins into adult female mosquitoes so that genetic changes can be passed on to their offspring. In this study, we examined how this approach performs in the yellow fever mosquito, *Aedes aegypti*. Our results show that gene editing proteins can reach the mosquito ovaries and induce heritable genetic changes when the ovarian targeting peptide is kept intact and combined with a compound that facilitates cellular uptake. When this targeting peptide is shortened, delivery to the ovaries becomes much less effective. We also observed that protein injection can influence mosquito reproduction, providing useful context for practical applications of this method. Overall, this work helps clarify how adult mosquito gene delivery is likely to be effective and outlines practical considerations for applying this technique in mosquito research and the development of genetic control strategies.

## 1. Introduction

Mosquito-borne infectious diseases continue to pose a significant challenge to global public health. As the primary vector for multiple arboviruses, *Aedes aegypti* (*Ae. aegypti*) mosquitoes represent a persistent threat to human health [1,2,3,4]. In recent years, the rapid advancement of gene editing technologies has provided powerful tools for investigating mosquito gene function and developing genetic control strategies [5]. However, the microinjection of embryos remains the most widely used delivery method in the field of mosquito gene editing [6]. This technique requires specialized experimental equipment and high-skilled operators, and is always associated with high embryo mortality rates. This limits its accessibility and scalability across different laboratory conditions [7].

Receptor-mediated ovary transduction of cargo (ReMOT) is a novel alternative gene editing strategy for mosquitoes. This method eliminates the need for embryo microinjection, enabling gene editing through simplified experimental operations [8]. The gene editing components are injected into adult female mosquitoes and are then transported via the hemolymph to the ovaries, entering the germline and achieving heritable genetic modification in offspring. Previous studies have demonstrated that fusing Cas9 with an ovary-targeting peptide enables ReMOT gene editing in multiple mosquito species [9,10]. Among these, the P2C peptide, which is derived from the yolk protein of *Drosophila melanogaster* (*D. melanogaster*), has been shown to enhance the uptake of Cas9 fusion protein in ovaries, exhibiting significant potential for application [11,12,13].

Despite the validation of the ReMOT technology mediated by P2C peptides in multiple mosquito species, the key design parameters for practical application have not been systematically evaluated. For example, it remains unclear whether the fusion configuration of Cas9-P2C protein affects delivery efficacy. In addition, although P2C is relatively short, there is a lack of experimental evidence to confirm whether its ovarian targeting activity persists in simplified or truncated peptide variants. Moreover, the potential impact of protein injection-based ReMOT operations on the mosquito reproductive capacity under standard experimental conditions requires further evaluation. Resolving these issues is essential to determining the applicability and practical value of the ReMOT technology.

Based on these considerations, we conducted a focused methodological evaluation of P2C-Cas9 fusion configurations in *Ae. aegypti*. We constructed Cas9 fusion proteins containing full-length and truncated P2C variants to assess their targeting capacity toward ovaries. Using the mosquito *kynurenine monooxygenase* (*KMO*) gene as a well-established and phenotypically traceable marker locus [14,15], we further evaluated protein expression characteristics, in vitro cleavage activity, and ReMOT-mediated gene editing efficacy. Additionally, we examined the reproductive performance of females after protein injection, providing practical reference for the application of ReMOT technology. Rather than aiming to maximize editing efficiency, our study focuses on defining key design parameters and methodological boundaries of the P2C-based ReMOT system in *Ae. aegypti*.

## 2. Materials and Methods

### 2.1. Mosquito Rearing

The *Ae. aegypti* mosquitoes used in this study were collected from Mengban, Yunnan Province, China, and continuously maintained under laboratory conditions. Mosquito rearing conditions were as follows: 27 ± 1 °C, relative humidity maintained at 75 ± 5% under 14 h light: 10 h dark. Larvae were reared in dechlorinated hatching water and fed fish food daily. Upon emergence, adult mosquitoes were transferred into cages and provided with 8% sucrose solution. All female mosquitoes used in gene editing and targeting experiments were 5 days post-eclosion.

### 2.2. Construction of Cas9-P2C Fusion Plasmid

Primers were designed using the *D. melanogaster* gene sequence (NM_078548) from the NCBI database. The P2C fragment was amplified (Vazyme, Nanjing, China) using cDNA obtained from *D. melanogaster* RNA by reverse transcription (Takara, Shiga, Japan) as the template and confirmed by sequencing (123 bp). To determine whether ovarian targeting activity depends on the structural integrity of P2C rather than discrete linear subregions, the full-length P2C sequence was divided into three equal-length truncated fragments (42 bp each), designated P2Ca, P2Cb, and P2Cc, to evaluate whether ovarian targeting requires the intact full-length sequence rather than isolated linear segments.

EGFP-tagged fusion fragments (P2C-EGFP, P2Ca-EGFP, P2Cb-EGFP, and P2Cc-EGFP) were firstly constructed for screening of ovarian targeting. Subsequently, using seamless cloning technology (Zomanbio, Beijing, China), these fragments were individually inserted into the pET-28b-Cas9-His expression vector to construct Cas9-P2C, Cas9-P2C-EGFP, Cas9-P2Ca-EGFP, Cas9-P2Cb-EGFP, and Cas9-P2Cc-EGFP expression plasmids. All constructs were validated by PCR and sequencing. The primer sequences used for construction are listed in Table 1, and plasmid sequences are provided in the Supplementary Materials.

### 2.3. Protein Expression and Purification

The constructed plasmid was transformed into *Escherichia coli* (*E. coli*) BL21(DE3) competent cells. When the bacterial culture absorbance reached a OD_600_ of approximately 0.4, isopropyl *β*-D-Thiogalactoside (IPTG) was added to a final concentration of 0.5 mM, and protein expression was induced overnight at 30 °C, 180 rpm. The overnight induced cells were sonicated and centrifuged, and the supernatant was collected for subsequent protein purification.

These expressed Cas9 fusion proteins were purified using a His-tag Protein Purification Kit (Beyotime, Shanghai, China). Briefly, the protein supernatant was incubated with His-affinity gel at 4 °C at 150 rpm for 2 h. Then, the mixture was transferred into a purification tube, and the bound proteins were sequentially eluted using washing buffer and imidazole-containing elution buffer. Each fraction was collected and analyzed by SDS-PAGE to assess protein purity.

To remove imidazole introduced during elution process, purified proteins were dialyzed using a 50 kDa molecular weight cutoff membrane (Solarbio, Beijing, China). The dialysis buffer contained 50 mM Tris-HCl (pH 8.0), 300 mM KCl, 0.1 mM ethylenediaminetetraacetic acid (EDTA), and 0.5 mM phenylmethylsulfonyl fluoride (PMSF). Following dialysis, these proteins were aliquoted and stored at −80 °C.

### 2.4. Protein Targeting Validation

Five days post-eclosion females were blood-fed with anticoagulated mouse blood (sodium heparin) for 1 h. At 24 h after blood feeding, engorged females were selected and then subjected to intrathoracic injection with Cas9-P2C-EGFP and other truncated fusion proteins (Cas9-P2Ca-EGFP, Cas9-P2Cb-EGFP, and Cas9-P2Cc-EGFP).

All proteins were injected at a concentration of 4000 ng/µL, with 200 nL injected per mosquito. Saponin (MCE) (final concentration 100 ng/µL) was added to the injection mixture in selected treatment groups. Cas9-EGFP served as the control. At 24 and 48 h post-injection, ovaries were dissected and EGFP fluorescence signals were detected using fluorescence microscopy to evaluate ovarian targeting efficiency of the different fusion proteins.

### 2.5. sgRNA Design and In Vitro Cleavage Validation

Based on the *Ae. aegypti KMO* gene sequence (LOC5571188) from the NCBI database, two sets of single-guide RNAs (sgRNAs) targeting the *KMO* exon were designed using the CHOPCHOP tool. All sgRNAs were synthesized by GentleGen Biotechnology Co., Ltd., Suzhou, China.

A *KMO* DNA fragment containing two target cleavage sites was first amplified by PCR (Vazyme, Nanjing, China). The in vitro cleavage activity of the designed *KMO* sgRNA_1_ and sgRNA_2_ was then evaluated by using the Cas9-P2C and Cas9-P2C-EGFP proteins, respectively, with a commercial Cas9 protein (Thermo Fisher Scientific, Waltham, MA, USA) serving as a control. Each reaction mixture contained 200 ng of DNA, sgRNA at a final concentration of 100 ng/µL, Cas9 protein at a final concentration of 150 ng/µL, and reaction buffer. After gentle mixing, reactions were incubated at 37 °C for 2 h, followed by the addition of proteinase K (Solarbio, Beijing, China) at a final concentration of 1 µg/µL, and incubated at 55 °C for 30 min. These reaction products were then subjected to 1% agarose gel electrophoresis to assess its cleavage activity.

### 2.6. ReMOT-Mediated Gene Editing and Genome Detection in Mosquitoes

ReMOT-mediated gene editing was similar to the targeted detection experimental protocol: 5 days post-eclosion wild-type females were fed mouse blood, and intrathoracic injection of 200 nL ribonucleoprotein (RNP) complexes was performed at 24 h post-blood feeding. Based on in vitro cleavage assay, the Cas9-P2C protein was selected to form RNP complexes with sgRNA_2_ for subsequent ReMOT gene editing.

RNP complexes were prepared by mixing Cas9-P2C protein at a final concentration of 4000 ng/µL, with sgRNA_2_ at 1000 ng/µL, and incubated at 25 °C for 20 min. Saponin (final concentration 100 ng/µL) was then added as an endosomal escape enhancer to facilitate cytosolic release of the delivered RNP complex following receptor-mediated endocytosis. Two days post-injection, mosquitoes were placed on moist oviposition paper for egg laying. After 5 days of egg development, we proceeded with standard hatching procedures.

After adult emergence, genomic DNA extraction was performed using the Phire™ Tissue Direct PCR Master Mix (Thermo Fisher Scientific, Waltham, MA, USA). Adult mosquitoes were mildly anesthetize with CO_2_, and a single leg was removed from each individual and placed into PCR tubes with 20 µL dilution buffer and 0.5 µL DNA release sequentially, then incubated tubes at 98 °C for 10 min. The supernatant in tubes was used as the DNA template for PCR amplification by *Ae. aegypti KMO* specific primers (Forward: AAATGCATAGCGAACCGTA, Reverse: CTTTTCGAGCAACATAAGAGC).

## 3. Results

### 3.1. Construction of P2C and Truncated Fragment Plasmids

**Figure 1:**
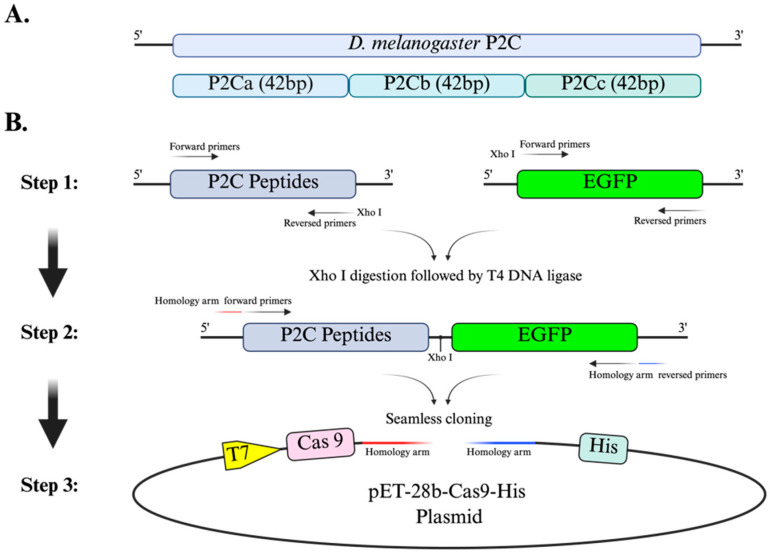
Schematic illustration of the construction of Cas9–P2C fusion expression plasmids. (**A**) Schematic representation of the full-length P2C peptide and truncated fragments. The P2C peptide was divided into three 42 bp segments (P2Ca, P2Cb, and P2Cc) for functional analysis. (**B**) The P2C/P2C small tags and EGFP fragments were first amplified using specific primers. The P2C-EGFP fusion fragment was generated through Xho I restriction digestion followed by T4 DNA ligase-mediated ligation. Subsequently, the fused fragment was inserted into the pET-28b-Cas9-His expression vector downstream of the Cas9 coding sequence using seamless cloning. The final construct places the Cas9-P2C (or Cas9-P2C-EGFP) fusion protein under the control of the T7 promoter, with a C-terminal 6 × His tag for protein purification (this figure was created with BioRender.com).

The P2C fragment was successfully amplified from cDNA reverse-transcribed from *D. melanogaster* RNA. Sequencing of the amplified product confirmed its identity with the reference sequence. To further assess the functional contribution of P2C subregions, the full-length P2C sequence was divided into three truncated fragments of 42 bp, named as P2Ca, P2Cb, and P2Cc (Figure 1A). All truncated fragments were successfully PCR amplified and verified by sequencing. Using an in vitro restriction enzyme and ligation strategy, we constructed P2C-EGFP, P2Ca-EGFP, P2Cb-EGFP, and P2Cc-EGFP fragments, and sequence verification confirmed their correctness.

These five fragments, P2C, P2C-EGFP, P2Ca-EGFP, P2Cb-EGFP, and P2Cc-EGFP, were subsequently cloned into the pET-28b-Cas9-His expression plasmid by seamless cloning (Figure 1B). PCR screening and sequencing confirmed the successful construction of all five Cas9 fusion plasmids (Supplementary Material S1).

### 3.2. Expression and Purification of Cas9 Fusion Proteins

Cas9-P2C and related fusion proteins were successfully expressed in *E. coli*, with protein bands detected at the expected molecular weights following IPTG induction. His-tag affinity purification effectively enriched the target proteins, and their identities were further confirmed by Western blot analysis using anti-Cas9 antibodies. Representative SDS-PAGE and Western blot results are provided in Supplementary Figures S1 and S2. Purified proteins were subsequently dialyzed to remove imidazole prior to downstream applications.

### 3.3. Ovarian Targeting of P2C and Truncated Peptides

**Figure 2:**
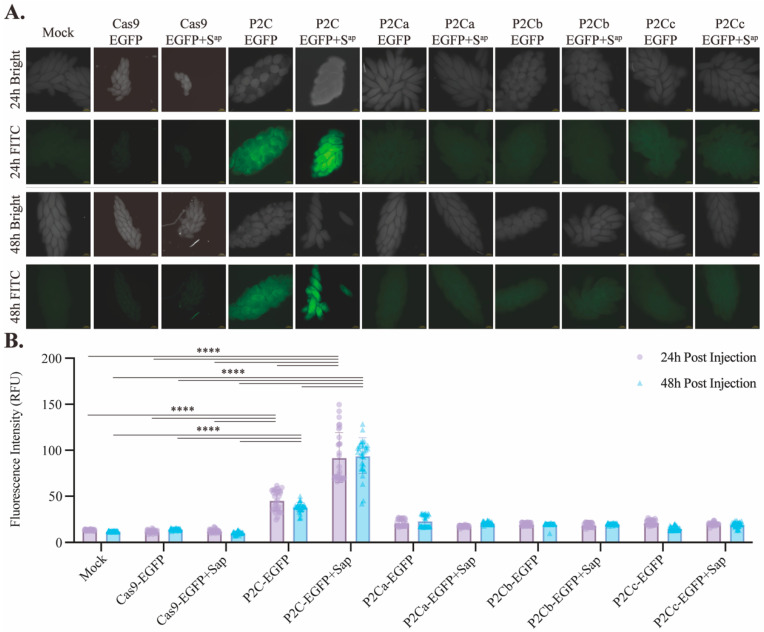
Ovarian Targeting of P2C and Truncated Peptides in *Ae. aegypti*. (**A**) Representative bright-field and fluorescence images of ovaries dissected at 24 h and 48 h post-injection are shown. No detectable EGFP fluorescence was observed in the mock and Cas9-EGFP control groups. Among the experimental treatments, ovarian fluorescence was detected in mosquitoes injected with Cas9-P2C-EGFP alone and Cas9-P2C-EGFP + saponin. In contrast, no detectable fluorescence signals were observed in ovaries injected with EGFP-tagged truncated P2C variants at both time points (S^ap^: saponin). (**B**) Quantification of ovarian fluorescence intensity (RFU) at 24 h and 48 h post-injection. Data are presented as mean ± SEM with individual data points shown (*n* = 30 per group). Statistical analysis was performed using two-way ANOVA followed by Tukey’s multiple comparisons test. Co-injection with saponin significantly increased fluorescence intensity compared with P2C-EGFP alone at both time points (****, *p* < 0.0001).

To assess the targeting ability of P2C and its three truncated peptides toward mosquito ovaries, EGFP-tagged Cas9 fusion proteins were injected intrathoracically into adult *Ae. aegypti* females, and ovarian fluorescence intensity was quantified at 24 h and 48 h post-injection (*n* = 30 per group). A significant accumulation of EGFP fluorescence was observed in ovaries following co-injection of Cas9-P2C-EGFP with saponin (Figure 2A). Quantitative analysis demonstrated that ovarian fluorescence intensity was significantly higher in the Cas9-P2C-EGFP + saponin group than in Cas9-EGFP and the mock control groups at 24 and 48 h post-injection (two-way ANOVA, *p* < 0.0001) (Figure 2B).

Injection of Cas9-P2C-EGFP alone also resulted in detectable ovarian fluorescence at both time points; however, the signal intensity was significantly lower than that observed in the saponin co-injection group (*p* < 0.0001) (Figure 2B).

In contrast, no appreciable EGFP signal was detected in ovaries from mosquitoes injected with Cas9-P2Ca-EGFP, Cas9-P2Cb-EGFP, or Cas9-P2Cc-EGFP at either 24 h or 48 h post-injection.

### 3.4. Effects of Protein Injection on Mosquito Reproductive Performance

To evaluate the potential physiological effects of protein injection, reproductive performance was assessed in female mosquitoes following injection. Analysis of egg production revealed significant differences among treatment groups (Welch’s one-way ANOVA, *p* < 0.05) (Table 2). Compared with the mock control, females injected with P2C-EGFP and P2C-EGFP + saponin showed a statistically significant reduction in egg production (Dunnett’s T3 test). In contrast, Cas9-EGFP, Cas9-EGFP + saponin, and P2Ca-EGFP did not significantly affect the number of eggs laid.

Egg hatching rates were further analyzed under the same rearing conditions. Consistent with the fecundity results, significant differences were detected in hatching rate among treatment groups (Kruskal–Wallis test, *p* < 0.0001). Dunn’s multiple comparisons test demonstrated that several treatment groups exhibited significantly reduced hatching rates compared with the mock control. Notably, the P2C-EGFP and P2C-EGFP + saponin groups showed the most pronounced reduction (*p* < 0.0001). Moderate but statistically significant reductions were also observed in the Cas9-EGFP, Cas9-EGFP + saponin, and P2Ca-EGFP groups (*p* < 0.01 or *p* < 0.001). In contrast, the P2Cb and P2Cc variants showed comparatively minor or inconsistent effects on hatching rate, despite reduced egg production in these groups.

### 3.5. In Vitro Cleavage Activity of Designed sgRNAs

**Figure 3:**
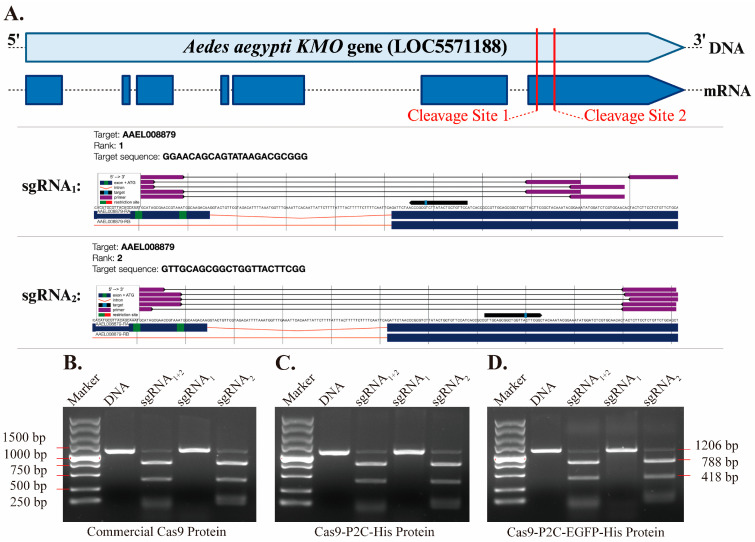
*KMO* sgRNA Design and In Vitro Cleavage Validation. (**A**) Schematic representation of sgRNA target sites and sequences within the *KMO* gene by CHOPCHOP. (**B**–**D**). In vitro cleavage assay mediated by different Cas9 proteins. We formed RNP complexes using (**B**) commercial Cas9, (**C**) Cas9-P2C-His, and (**D**) Cas9-P2C-EGFP-His proteins with sgRNA_1_ and sgRNA_2_, to cleave the in vitro amplified *KMO* DNA fragment (Marker: DNA marker; DNA: *KMO* DNA only; sgRNA_1+2_: *KMO* DNA + sgRNA_1_ + sgRNA_2_ + Cas9; sgRNA_1_: *KMO* DNA + sgRNA_1_ + Cas9; sgRNA_2_: *KMO* DNA + sgRNA_2_ + Cas9) (this figure was created with BioRender.com).

Two sgRNAs targeting the exonic regions of the *Ae. aegypti KMO* gene were designed and synthesized, and the sgRNA sequences and predicted cleavage sites are shown (Figure 3A). To evaluate the nuclease activity of Cas9-P2C fusion proteins, an amplified *KMO* fragment containing two target sites was purified for in vitro cleavage assays. Agarose gel electrophoresis revealed that both Cas9-P2C and Cas9-P2C-EGFP fusion proteins efficiently cleaved the target fragment, comparable to the activity observed with a commercial Cas9 protein (Figure 3B–D). However, among these two synthesized sgRNAs, only sgRNA_2_ guided Cas9 protein to cleave the fragment, whereas sgRNA_1_ did not achieve cleavage under the same conditions.

### 3.6. ReMOT-Mediated Gene Editing of the KMO in Ae. aegypti

**Figure 4:**
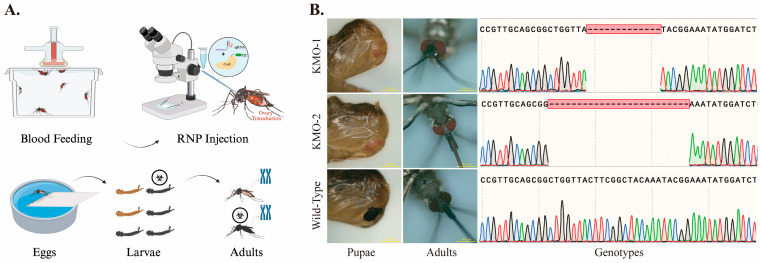
ReMOT-mediated gene editing of the *KMO* gene in *Ae. aegypti*. (**A**) Schematic overview of the ReMOT gene editing strategy using Cas9-P2C protein in combination with sgRNA_2_. (**B**) Representative phenotypes of pupae and adults following ReMOT-mediated editing, together with corresponding sanger sequencing chromatograms of the *KMO* target region. Sequence analysis of TA-cloned PCR products derived from edited individuals, revealing 13 bp and 25 bp deletion at the *KMO* target locus, whereas wild-type mosquitoes display clean sequencing traces (this figure was created with BioRender.com).

Based on in vitro cleavage results, the Cas9-P2C protein combined with sgRNA_2_ was selected for ReMOT gene editing (Figure 4A). A total of 20 female mosquitoes were injected, with 12 surviving post-injection and producing 314 eggs. These eggs were incubated using the previously described protocol, resulting in 149 hatched larvae (hatching rate: 47.2%) and 136 emerged adults (emergence rate: 91.3%).

Genomic DNA was extracted from the legs of emerged adults and performed PCR amplification and sequencing of DNA containing the sgRNA_2_ cleavage site. Sequencing results revealed that, among the 136 sequenced samples, two exhibited overlapping peaks at the cleavage sites, indicating gene-edited individuals. Further TA-cloning and sequencing of these PCR products confirmed the presence of a genotype with 13 bp and 25 bp deletion (Figure 4B), demonstrating successful gene editing of the mosquito *KMO* gene using the ReMOT technology and the generation of *KMO* gene chimeric mutant mosquitoes.

## 4. Discussion

ReMOT technology provides a practical alternative to embryonic microinjection for mosquito genome editing; however, its performance is influenced by various designs and operational parameters that remain incompletely characterized. This study experimentally evaluated the application of the ReMOT CRISPR/Cas9 system based on the *D. melanogaster* P2C peptide in *Ae. aegypti*, with a primary focus on methodological evaluation. By constructing and expressing Cas9-P2C fusion proteins with different configurations, combined with the results of ovarian targeting analyses, in vitro cleavage assays, and in vivo gene editing validation, we have established several key design considerations and boundaries for the practical application of the P2C-ReMOT gene editing system in mosquitoes.

Previous ReMOT studies primarily focused on establishing gene editing methods and extending ReMOT to other species [16,17,18,19]. In contrast, our study provides a focused comparative assessment of the P2C delivery system, elucidating constraints related to peptide integrity, fusion configuration, and endosomal escape requirements. Rather than treating P2C as a constant ovarian targeting element, we directly investigated its functional limitations through targeted modification of fusion protein configuration. Results demonstrate that the full-length P2C peptide is essential for maintaining ovarian targeting capability, while linear truncation leads to a significant function loss, indicating that ovarian targeting is highly dependent on P2C structural integrity. At the same time, the full-length Cas9-P2C fusion proteins retained inherent nuclease activity and supported ReMOT-mediated gene editing in *Ae. aegypti*. In summary, these findings clarify important design considerations influencing the performance of P2C-based ReMOT, providing practical references for the standardization and further optimization of receptor-mediated delivery strategy in mosquitoes.

It is important to note that the Cas9-P2C fusion configuration employed in this study differs from the previously reported N-terminal P2C fusion strategy [8]. The C-terminal P2C fusion structure adopted in this study maintains Cas9 nuclease activity while enabling ReMOT-mediated gene editing. It is acknowledged that the efficiency of ReMOT could be influenced by multiple factors, including the fusion configuration between Cas9 and P2C, which may impact delivery and editing efficiency [20]. Therefore, the primary focus of this study describes the results and characteristics obtained under the Cas9-P2C fusion scheme, providing references for future comparisons aimed at further optimizing fusion configurations and delivery efficiency.

In ovarian targeting experiments, it was observed that the P2C-Cas9-EGFP fusion protein exhibited detectable fluorescence enrichment in *Ae. aegypti* ovaries both in the presence and absence of saponin, indicating that P2C-mediated ovarian uptake can occur independently of endosomal escape enhancement. However, co-injection with saponin resulted in markedly stronger and more clearly defined fluorescence signals, suggesting improved intracellular availability of the delivered cargo. In the P2C-mediated ReMOT system, the Cas9 RNP complex enters the oocyte via yolk protein receptor-mediated endocytosis. Receptor-mediated endocytosis generally functions to transport cargo into the endosomal–lysosomal pathway. Without effective release from endosomes, the entry of exogenous cargoes into the cytoplasm and subsequent access to the nucleus to exert their function is limited [21,22]. Consequently, endosomal escape is regarded as a major limiting step for achieving heritable gene editing via ReMOT.

Saponins are a class of membrane active triterpenoid compounds that are widely used as endosomal escape enhancers in cellular delivery studies. Previous studies have shown that saponins can temporarily increase membrane permeability through interactions with sterol-rich endosomal membranes, thereby facilitating the release of endocytosed protein or nucleic acid cargo into the cytoplasm [23]. Within the ReMOT system, the introduction of saponin does not contribute to the targeting of ovaries or oocytes. Instead, it facilitates the release of cargo from the endosomes after successful endocytosis, serving as a critical condition for achieving effective gene editing.

In mosquito gene editing experiments, we successfully generated *KMO*-edited chimeric individuals in *Ae. aegypti* using the ReMOT strategy based on the Cas9-P2C fusion protein, demonstrating the feasibility of this strategy under the conditions of this study. However, the ReMOT gene editing protocol could be influenced by multiple factors, including protein delivery efficiency, sgRNA activity, target gene site characteristics, and individual physiological status. This study was not designed to maximize editing efficiency; instead, it focused on feasibility validation and defining methodological boundaries. Therefore, the results of this study should primarily be interpreted as an assessment of the applicability and limitations of the P2C-ReMOT system in *Ae. aegypti*, rather than a systematic determination of ReMOT strategy’s performance ceiling.

Additionally, this study evaluated the reproductive capacity of *Ae. aegypti* mosquitoes following protein injection. Under the experimental conditions in this study, protein injection was significantly associated with changes in reproductive performance. Specifically, P2C-based protein delivery was associated with reduced egg production and decreased hatching rates, particularly in the P2C-EGFP and P2C-EGFP + saponin groups. These observations provide additional insight into the potential physiological responses of mosquitoes during protein delivery in the gene editing process. From the application perspective, these reproductive effects emphasize the importance of carefully considering parameters such as delivery dosage, fusion configuration, and operational procedures when using the ReMOT strategy. Incorporating these factors into experimental design may improve the consistency, scalability, and reproducibility of ReMOT technology in mosquitoes and other arthropods.

## 5. Conclusions

Overall, this study does not aim to establish a gene editing strategy superior to existing methods. Instead, we provide a comparative evaluation of selected design parameters to further clarify the applicability and limitations of the P2C-based ReMOT-Cas9 system in *Ae. aegypti*. The study emphasizes the importance of P2C structural integrity, variations in delivery systems, and the role of endosomal escape enhancement in achieving functional gene editing. These findings provide methodological insights for further application and the optimization of ReMOT technology in mosquito gene function research and genetic control strategies.

## Figures and Tables

**Table 1:** The sequences of primers.

Primer (s)	Sequence (s) (5′-3′)
P2C Forward	AATCTGCAGCAGCAGCGC
P2C Reversed	CTCGAGGTTCTTAACCTCCTCGCTGTAGTCC
P2Ca Forward	AATCTGCAGCAGCAGCGC
P2Ca Reversed	CTCGAGCTGGTTGCCGTTCTTGCCG
P2Cb Forward	GACTACCAGGATCAGAGC
P2Cb Reversed	CTCGAGCCTCTGGTTCTTCCTCT
P2Cc Forward	GAAGAGGAAGGTGGCTAGGACCAGCAGCGAG
P2Cc Reversed	TGCTCGAGTGCGGCCGCTTTGTACAGCTCGTC
EGFP Forward	CTCGAGTACAGTGGGGGTGGAGGC
EGFP Reversed	GCGGCCGCTTTGTACAGCTCGTCCATACCCAG

**Table 2:** Reproductive performance of *Ae. aegypti* following protein injection.

Group	Egg Number (Mean ± SD)	Hatching Rate (Mean ± SD)
Mock	35.03 ± 8.47	0.894 ± 0.044
Cas9-EGFP	32.73 ± 5.06	0.734 ± 0.060
Cas9-EGFP + Sap	31.80 ± 6.83	0.671 ± 0.054
P2C-EGFP	28.37 ± 5.79	0.336 ± 0.057
P2C-EGFP + Sap	26.50 ± 5.21	0.365 ± 0.038
P2Ca-EGFP	32.20 ± 6.59	0.632 ± 0.047
P2Ca-EGFP + Sap	27.83 ± 5.50	0.619 ± 0.078
P2Cb-EGFP	26.77 ± 4.39	0.703 ± 0.080
P2Cb-EGFP + Sap	26.93 ± 3.60	0.701 ± 0.128
P2Cc-EGFP	27.40 ± 4.55	0.657 ± 0.064
P2Cc-EGFP + Sap	26.83 ± 4.65	0.707 ± 0.072

Data are presented as mean ± SD (*n* = 30 females per group). Egg numbers were analyzed by Welch’s one-way ANOVA followed by Dunnett’s T3 multiple comparisons test, with the mock as the control. Hatching rate was calculated for each individual as larvae/eggs, and differences among groups were analyzed using the Kruskal–Wallis test followed by Dunn’s multiple comparisons test, with the mock group as the control.

## Data Availability

The data presented in this study will be available on request.

## Supplementary Materials

The following supporting information can be downloaded at: https://www.mdpi.com/article/10.3390/insects17050451/s1, Supplementary Materials S1: The sequences of pET-28b-Cas9-His, P2C, P2C-EGFP, P2Ca-EGFP, P2Cb-EGFP, and P2Cc-EGFP; Supplementary Materials S2: Cas9 fusion protein expression and Western blot validation; Supplementary Materials S3: Cas9 fusion protein purification and dialysis.
