# Supplementary material for: Methodological Evaluation of a P2C-Based ReMOT CRISPR/Cas9 System in Aedes aegypti
Source: Insects. 2026 Apr 24;17(5):451. doi: 10.3390/insects17050451 (PMC13207695; doi:10.3390/insects17050451)
Supplement: Supplementary file 1 [file insects-17-00451-s001.zip › Supplementary Materials S1-S3.pdf]

Supplementary Materials S1

1. The sequences of pET-28b-Cas9-His Plasmid:

(represent the Cas9 sequence.)

AGCGCCTGATGCGGTATTTTCTCCTTACGCATCTGTGCGGTATTTCACACCGCAATGGTG
CACTCTCAGTACAATCTGCTCTGATGCCGCATAGTTAAGCCAGTATACACTCCGCTATCG
CTACGTGACTGGGTCATGGCTGCGCCCCGACACCCGCCAACACCCGCTGACGCGCCCT
GACGGGCTTGTCTGCTCCCGGCATCCGCTTACAGACAAGCTGTGACCGTCTCCGGGAG
CTGCATGTGTCAGAGGTTTTCACCGTCATCACCGAAACGCGCGAGGCAGCTGCGGTAA
AGCTCATCAGCGTGGTCGTGAAGCGATTCACAGATGTCTGCCTGTTCATCCGCGTCCA
GCTCGTTGAGTTTCTCCAGAAGCGTTAATGTCTGGCTTCTGATAAAGCGGGCCATGTTA
AGGGCGGTTTTTTCCTGTTTGGTCACTGATGCCTCCGTGTAAGGGGGATTTCTGTTCAT
GGGGGTAATGATACCGATGAAACGAGAGAGGATGCTCACGATACGGGTTACTGATGAT
GAACATGCCCGGTTACTGGAACGTTGTGAGGGTAAACAACTGGCGGTATGGATGCGGC
GGGACCAGAGAAAAATCACTCAGGGTCAATGCCAGCGCTTCGTTAATACAGATGTAGG
TGTTCCACAGGGTAGCCAGCAGCATCCTGCGATGCAGATCCGGAACATAATGGTGCAG
GGCGCTGACTTCCGCGTTTCCAGACTTTACGAAACACGGAAACCGAAGACCATTCATG
TTGTTGCTCAGGTCGCAGACGTTTTGCAGCAGCAGTCGCTTCACGTTCGCTCGCGTATC
GGTGATTCATTCTGCTAACCAGTAAGGCAACCCCGCCAGCCTAGCCGGGTCCTCAACG
ACAGGAGCACGATCATGCGCACCCGTGGGGCCGCCATGCCGGCGATAATGGCCTGCTT
CTCGCCGAAACGTTTGGTGGCGGGACCAGTGACGAAGGCTTGAGCGAGGGCGTGCAA
GATTCCGAATACCGCAAGCGACAGGCCGATCATCGTCGCGCTCCAGCGAAAGCGGTCC
TCGCCGAAAATGACCCAGAGCGCTGCCGGCACCTGTCCTACGAGTTGCATGATAAAGA
AGACAGTCATAAGTGCGGCGACGATAGTCATGCCCCGCGCCCACCGGAAGGAGCTGA
CTGGGTTGAAGGCTCTCAAGGGCATCGGTCGAGATCCCGGTGCCTAATGAGTGAGCTA
ACTTACATTAATTGCGTTGCGCTCACTGCCCGCTTTCCAGTCGGGAAACCTGTCGTGCC
AGCTGCATTAATGAATCGGCCAACGCGCGGGGAGAGGCGGTTTGCGTATTGGGCGCCA
GGGTGGTTTTTCTTTTCACCAGTGAGACGGGCAACAGCTGATTGCCCTTCACCGCCTG
GCCCTGAGAGAGTTGCAGCAAGCGGTCCACGCTGGTTTGCCCCAGCAGGCGAAAATC
CTGTTTGATGGTGGTTAACGGCGGGATATAACATGAGCTGTCTTCGGTATCGTCGTATCC
CACTACCGAGATATCCGCACCAACGCGCAGCCCGGACTCGGTAATGGCGCGCATTGCG
CCCAGCGCCATCTGATCGTTGGCAACCAGCATCGCAGTGGGAACGATGCCCTCATTCA
GCATTTGCATGGTTTGTTGAAAACCGGACATGGCACTCCAGTCGCCTTCCCGTTCCGCT
ATCGGCTGAATTTGATTGCGAGTGAGATATTTATGCCAGCCAGCCAGACGCAGACGCG
CCGAGACAGAACTTAATGGGCCCGCTAACAGCGCGATTTGCTGGTGACCCAATGCGAC
CAGATGCTCCACGCCCAGTCGCGTACCGTCTTCATGGGAGAAAATAATACTGTTGATGG
GTGTCTGGTCAGAGACATCAAGAAATAACGCCGGAACATTAGTGCAGGCAGCTTCCAC
AGCAATGGCATCCTGGTCATCCAGCGGATAGTTAATGATCAGCCCACTGACGCGTTGCG
CGAGAAGATTGTGCACCGCCGCTTTACAGGCTTCGACGCCGCTTCGTTCTACCATCGA
CACCACCACGCTGGCACCCAGTTGATCGGCGCGAGATTTAATCGCCGCGACAATTTGC
GACGGCGCGTGCAGGGCCAGACTGGAGGTGGCAACGCCAATCAGCAACGACTGTTTG
CCCGCCAGTTGTTGTGCCACGCGGTTGGGAATGTAATTCAGCTCCGCCATCGCCGCTTC
CACTTTTTCCCGCGTTTTCGCAGAAACGTGGCTGGCCTGGTTCACCACGCGGGAAACG
GTCTGATAAGAGACACCGGCATACTCTGCGACATCGTATAACGTTACTGGTTTCACATT

CACCACCCTGAATTGACTCTCTTCCGGGCGCTATCATGCCATACCGCGAAAGGTTTTGC
GCCATTCGATGGTGTCCGGGATCTCGACGCTCTCCCTTATGCGACTCCTGCATTAGGAA
GCAGCCCAGTAGTAGGTTGAGGCCGTTGAGCACCGCCGCCGCAAGGAATGGTGCATG
CAAGGAGATGGCGCCCAACAGTCCCCCGGCCACGGGGCCTGCCACCATACCCACGCC
GAAACAAGCGCTCATGAGCCCGAAGTGGCGAGCCCGATCTTCCCCATCGGTGATGTCG
GCGATATAGGCGCCAGCAACCGCACCTGTGGCGCCGGTGATGCCGGCCACGATGCGTC
CGGCGTAGAGGATCGAGATCTCGATCCCGCGAAATTAATACGACTCACTATAGGGGAAT
TGTGAGCGGATAACAATTCCCCTCTAGAAATAATTTTGTTTAACTTTAAGAAGGAGATAT
ACCATGGACAAGAAGTACTCCATTGGGCTCGATATCGGCACAAACAGCGTCGGCTGGG
CCGTCATTACGGACGAGTACAAGGTGCCGAGCAAAAAATTCAAAGTTCTGGGCAATAC
CGATCGCCACAGCATAAAGAAGAACCTCATTGGCGCCCTCCTGTTCGACTCCGGGGAG
ACGGCCGAAGCCACGCGGCTCAAAAGAACAGCACGGCGCAGATATACCCGCAGAAAG
AATCGGATCTGCTACCTGCAGGAGATCTTTAGTAATGAGATGGCTAAGGTGGATGACTC
TTTCTTCCATAGGCTGGAGGAGTCCTTTTTGGTGGAGGAGGATAAAAAGCACGAGCGC
CACCCAATCTTTGGCAATATCGTGGACGAGGTGGCGTACCATGAAAAGTACCCAACCA
TATATCATCTGAGGAAGAAGCTTGTAGACAGTACTGATAAGGCTGACTTGCGGTTGATC
TATCTCGCGCTGGCGCATATGATCAAATTTCGGGGACACTTCCTCATCGAGGGGGACCT
GAACCCAGACAACAGCGATGTCGACAAACTCTTTATCCAACTGGTTCAGACTTACAAT
CAGCTTTTCGAAGAGAACCCGATCAACGCATCCGGAGTTGACGCCAAAGCAATCCTGA
GCGCTAGGCTGTCCAAATCCCGGCGGCTCGAAAACCTCATCGCACAGCTCCCTGGGGA
GAAGAAGAACGGCCTGTTTGGTAATCTTATCGCCCTGTCACTCGGGCTGACCCCCAAC
TTTAAATCTAACTTCGACCTGGCCGAAGATGCCAAGCTTCAACTGAGCAAAGACACCT
ACGATGATGATCTCGACAATCTGCTGGCCCAGATCGGCGACCAGTACGCAGACCTTTTT
TTGGCGGCAAAGAACCTGTCAGACGCCATTCTGCTGAGTGATATTCTGCGAGTGAACA
CGGAGATCACCAAAGCTCCGCTGAGCGCTAGTATGATCAAGCGCTATGATGAGCACCA
CCAAGACTTGACTTTGCTGAAGGCCCTTGTCAGACAGCAACTGCCTGAGAAGTACAA
GGAAATTTTCTTCGATCAGTCTAAAAATGGCTACGCCGGATACATTGACGGCGGAGCA
AGCCAGGAGGAATTTTACAAATTTATTAAGCCCATCTTGGAAAAAATGGACGGCACCG
AGGAGCTGCTGGTAAAGCTTAACAGAGAAGATCTGTTGCGCAAACAGCGCACTTTCG
ACAATGGAAGCATCCCCCACCAGATTCACCTGGGCGAACTGCACGCTATCCTCAGGCG
GCAAGAGGATTTCTACCCCTTTTTGAAAGATAACAGGGAAAAGATTGAGAAAATCCTC
ACATTTCGGATACCCTACTATGTAGGCCCCCTCGCCCGGGGAAATTCCAGATTCGCGTG
GATGACTCGCAAATCAGAAGAGACCATCACTCCCTGGAACTTCGAGGAAGTCGTGGAT
AAGGGGGCCTCTGCCCAGTCCTTCATCGAAAGGATGACTAACTTTGATAAAAATCTGC
CTAACGAAAAGGTGCTTCCTAAACACTCTCTGCTGTACGAGTACTTCACAGTTTATAAC
GAGCTCACCAAGGTCAAATACGTCACAGAAGGGATGAGAAAGCCAGCATTCCTGTCT
GGAGAGCAGAAGAAAGCTATCGTGGACCTCCTCTTCAAGACGAACCGGAAAGTTACC
GTGAAACAGCTCAAAGAAGACTATTTCAAAAAGATTGAATGTTTCGACTCTGTTGAAA
TCAGCGGAGTGGAGGATCGCTTCAACGCATCCCTGGGAACGTATCACGATCTCCTGAA
AATCATTAAAGACAAGGACTTCCTGGACAATGAGGAGAACGAGGACATTCTTGAGGA
CATTGTCCTCACCCTTACGTTGTTTGAAGATAGGGAGATGATTGAAGAACGCTTGAAA
ACTTACGCTCATCTCTTCGACGACAAAGTCATGAAACAGCTCAAGAGGCGCCGATATA
CAGGATGGGGGCGGCTGTCAAGAAAACTGATCAATGGGATCCGAGACAAGCAGAGTG
GAAAGACAATCCTGGATTTTCTTAAGTCCGATGGATTTGCCAACCGGAACTTCATGCAG

TTGATCCATGATGACTCTCTCACCTTTAAGGAGGACATCCAGAAAGCACAAGTTTCTGG
CCAGGGGGACAGTCTTCACGAGCACATCGCTAATCTTGCAGGTAGCCCAGCTATCAAA
AAGGGAATACTGCAGACCGTTAAGGTCGTGGATGAACTCGTCAAAGTAATGGGAAGG
CATAAGCCCGAGAATATCGTTATCGAGATGGCCCGAGAGAACCAAACTACCCAGAAGG
GACAGAAGAACAGTAGGGAAAGGATGAAGAGGATTGAAGAGGGTATAAAAGAACTG
GGGTCCCAAATCCTTAAGGAACACCCAGTTGAAAACACCCAGCTTCAGAATGAGAAG
CTCTACCTGTACTACCTGCAGAACGGCAGGGACATGTACGTGGATCAGGAACTGGACA
TCAATCGGCTCTCCGACTACGACGTGGATCATATCGTGCCCCAGTCTTTTCTCAAAGAT
GATTCTATTGATAATAAAGTGTTGACAAGATCCGATAAAAATAGAGGGAAGAGTGATAA
CGTCCCCTCAGAAGAAGTTGTCAAGAAAATGAAAAATTATTGGCGGCAGCTGCTGAAC
GCCAAACTGATCACACAACGGAAGTTCGATAATCTGACTAAGGCTGAACGAGGTGGCC
TGTCTGAGTTGGATAAAGCCGGCTTCATCAAAAGGCAGCTTGTTGAGACACGCCAGAT
CACCAAGCACGTGGCCCAAATTCTCGATTCACGCATGAACACCAAGTACGATGAAAAT
GACAAACTGATTCGAGAGGTGAAAGTTATTACTCTGAAGTCTAAGCTGGTCTCAGATTT
CAGAAAGGACTTTCAGTTTTATAAGGTGAGAGAGATCAACAATTACCACCATGCGCAT
GATGCCTACCTGAATGCAGTGGTAGGCACTGCACTTATCAAAAAATATCCCAAGCTTGA
ATCTGAATTTGTTTACGGAGACTATAAAGTGTACGATGTTAGGAAAATGATCGCAAAGT
CTGAGCAGGAAATAGGCAAGGCCACCGCTAAGTACTTCTTTTACAGCAATATTATGAAT
TTTTTCAAGACCGAGATTACACTGGCCAATGGAGAGATTCGGAAGCGACCACTTATCG
AAACAAACGGAGAAACAGGAGAAATCGTGTGGGACAAGGGTAGGGATTTCGCGACA
GTCCGGAAGGTCCTGTCCATGCCGCAGGTGAACATCGTTAAAAAGACCGAAGTACAG
ACCGGAGGCTTCTCCAAGGAAAGTATCCTCCCGAAAAGGAACAGCGACAAGCTGATC
GCACGCAAAAAAGATTGGGACCCCAAGAAATACGGCGGATTCGATTCTCCTACAGTCG
CTTACAGTGTACTGGTTGTGGCCAAAGTGGAGAAAGGGAAGTCTAAAAAACTCAAAA
GCGTCAAGGAACTGCTGGGCATCACAATCATGGAGCGATCAAGCTTCGAAAAAAACC
CCATCGACTTTCTCGAGGCGAAAGGATATAAAGAGGTCAAAAAAGACCTCATCATTAA
GCTTCCCAAGTACTCTCTCTTTGAGCTTGAAAACGGCCGGAAACGAATGCTCGCTAGT
GCGGGCGAGCTGCAGAAAGGTAACGAGCTGGCACTGCCCTCTAAATACGTTAATTTCT
TGTATCTGGCCAGCCACTATGAAAAGCTCAAAGGGTCTCCCGAAGATAATGAGCAGAA
GCAGCTGTTCGTGGAACAACACAAACACTACCTTGATGAGATCATCGAGCAAATAAGC
GAATTCTCCAAAAGAGTGATCCTCGCCGACGCTAACCTCGATAAGGTGCTTTCTGCTTA
CAATAAGCACAGGGATAAGCCCATCAGGGAGCAGGCAGAAAACATTATCCACTTGTTT
ACTCTGACCAACTTGGGCGCGCCTGCAGCCTTCAAGTACTTCGACACCACCATAGACA
GAAAGCGGTACACCTCTACAAAGGAGGTCCTGGACGCCACACTGATTCATCAGTCAAT
TACGGGGCTCTATGAAACAAGAATCGACCTCTCTCAGCTCGGTGGAGACAGCAGGGCT
GACCCCAAGAAGAAGAGGAAGGTGGCGGCCGCACTCGAGCACCACCACCACCACCA
CTGAGATCCGGCTGCTAACAAAGCCCGAAAGGAAGCTGAGTTGGCTGCTGCCACCGC
TGAGCAATAACTAGCATAACCCCTTGGGGCCTCTAAACGGGTCTTGAGGGGTTTTTTGC
TGAAAGGAGGAACTATATCCGGATTGGCGAATGGGACGCGCCCTGTAGCGGCGCATTA
AGCGCGGCGGGTGTGGTGGTTACGCGCAGCGTGACCGCTACACTTGCCAGCGCCCTAG
CGCCCGCTCCTTTCGCTTTCTTCCCTTCCTTTCTCGCCACGTTCGCCGGCTTTCCCCGTC
AAGCTCTAAATCGGGGGCTCCCTTTAGGGTTCCGATTTAGTGCTTTACGGCACCTCGAC
CCCAAAAAACTTGATTAGGGTGATGGTTCACGTAGTGGGCCATCGCCCTGATAGACGG
TTTTTCGCCCTTTGACGTTGGAGTCCACGTTCTTTAATAGTGGACTCTTGTTCCAAACT

GGAACAACACTCAACCCTATCTCGGTCTATTCTTTTGATTTATAAGGGATTTTGCCGATT
TCGGCCTATTGGTTAAAAAATGAGCTGATTTAACAAAAATTTAACGCGAATTTTAACAA
AATATTAACGCTTACAATTTAGGTGGCACTTTTCGGGGAAATGTGCGCGGAACCCCTAT
TTGTTTATTTTTCTAAATACATTCAAATATGTATCCGCTCATGAATTAATTCTTAGAAAAA
CTCATCGAGCATCAAATGAAACTGCAATTTATTCATATCAGGATTATCAATACCATATTTT
TGAAAAAGCCGTTTCTGTAATGAAGGAGAAAACTCACCGAGGCAGTTCCATAGGATGG
CAAGATCCTGGTATCGGTCTGCGATTCCGACTCGTCCAACATCAATACAACCTATTAATT
TCCCCTCGTCAAAAATAAGGTTATCAAGTGAGAAATCACCATGAGTGACGACTGAATC
CGGTGAGAATGGCAAAAGTTTATGCATTTCTTTCCAGACTTGTTCAACAGGCCAGCCAT
TACGCTCGTCATCAAAATCACTCGCATCAACCAAACCGTTATTCATTCGTGATTGCGCC
TGAGCGAGACGAAATACGCGATCGCTGTTAAAAGGACAATTACAAACAGGAATCGAAT
GCAACCGGCGCAGGAACACTGCCAGCGCATCAACAATATTTTCACCTGAATCAGGATA
TTCTTCTAATACCTGGAATGCTGTTTTCCCGGGGATCGCAGTGGTGAGTAACCATGCAT
CATCAGGAGTACGGATAAAATGCTTGATGGTCGGAAGAGGCATAAATTCCGTCAGCCA
GTTTAGTCTGACCATCTCATCTGTAACATCATTGGCAACGCTACCTTTGCCATGTTTCAG
AAACAACTCTGGCGCATCGGGCTTCCCATACAATCGATAGATTGTCGCACCTGATTGCC
CGACATTATCGCGAGCCCATTTATACCCATATAAATCAGCATCCATGTTGGAATTTAATCG
CGGCCTAGAGCAAGACGTTTCCCGTTGAATATGGCTCATAACACCCCTTGTATTACTGT
TTATGTAAGCAGACAGTTTTATTGTTCATGACCAAAATCCCTTAACGTGAGTTTTCGTTC
CACTGAGCGTCAGACCCCGTAGAAAAGATCAAAGGATCTTCTTGAGATCCTTTTTTTCT
GCGCGTAATCTGCTGCTTGCAAACAAAAAAACCACCGCTACCAGCGGTGGTTTGTTTG
CCGGATCAAGAGCTACCAACTCTTTTTCCGAAGGTAACTGGCTTCAGCAGAGCGCAGA
TACCAAATACTGTCCTTCTAGTGTAGCCGTAGTTAGGCCACCACTTCAAGAACTCTGTA
GCACCGCCTACATACCTCGCTCTGCTAATCCTGTTACCAGTGGCTGCTGCCAGTGGCGA
TAAGTCGTGTCTTACCGGGTTGGACTCAAGACGATAGTTACCGGATAAGGCGCAGCGG
TCGGGCTGAACGGGGGGTTCGTGCACACAGCCCAGCTTGGAGCGAACGACCTACACC
GAACTGAGATACCTACAGCGTGAGCTATGAGAAAGCGCCACGCTTCCCGAAGGGAGA
AAGGCGGACAGGTATCCGGTAAGCGGCAGGGTCGGAACAGGAGAGCGCACGAGGGA
GCTTCCAGGGGGAAACGCCTGGTATCTTTATAGTCCTGTCGGGTTTCGCCACCTCTGAC
TTGAGCGTCGATTTTTGTGATGCTCGTCAGGGGGGCGGAGCCTATGGAAAAACGCCAG
CAACGCGGCCTTTTTACGGTTCCTGGCCTTTTGCTGGCCTTTTGCTCACATGTTCTTTCC
TGCGTTATCCCCTGATTCTGTGGATAACCGTATTACCGCCTTTGAGTGAGCTGATACCGC
TCGCCGCAGCCGAACGACCGAGCGCAGCGAGTCAGTGAGCGAGGAAGCGGAAG

2. The sequences of P2C:

(represent the P2C sequence.)

AATCTGCAGCAGCAGCGCCAGCACGGCAAGAACGGCAACCAGGACTACCAGGATCAG
AGCAACGAACAGAGGAAGAACCAGAGGACCAGCAGCGAGGAGGACTACAGCGAGG
AGGTTAAGAAC

3. The sequences of P2C-EGFP:

(represent the P2C sequence; represent the EGFP sequence.)

AATCTGCAGCAGCAGCGCCAGCACGGCAAGAACGGCAACCAGGACTACCAGGATCAG
AGCAACGAACAGAGGAAGAACCAGAGGACCAGCAGCGAGGAGGACTACAGCGAGG

AGGTTAAGAACCTCGAGTACAGTGGGGGTGGAGGCTCTATGGTTAGCAAGGGCGAGG
AACTGTTCACCGGCGTGGTTCCGATTCTGGTGGAACTGGACGGTGATGTTAACGGCCA
CAAATTTAGCGTGAGCGGCGAAGGCGAGGGCGATGCGACCTATGGTAAACTGACCCTG
AAGTTCATCTGCACCACCGGTAAACTGCCGGTGCCGTGGCCGACCCTGGTTACCACCC
TGACCTACGGTGTGCAATGCTTCAGCCGTTATCCGGACCACATGAAACAGCACGATTTC
TTTAAGAGCGCGATGCCGGAAGGTTACGTTCAAGAGCGTACCATTTTCTTTAAAGACG
ATGGCAACTATAAGACCCGTGCGGAAGTTAAATTCGAGGGTGACACCCTGGTGAACCG
TATCGAACTGAAAGGCATTGACTTTAAAGAGGACGGTAACATCCTGGGCCACAAACTG
GAATACAACTATAACAGCCACAACGTTTACATCATGGCGGATAAACAAAAGAACGGCA
TTAAAGTGAACTTTAAGATCCGTCACAACATTGAGGACGGTAGCGTTCAGCTGGCGGA
TCACTACCAGCAAAACACCCCGATTGGTGACGGCCCGGTTCTGCTGCCGGATAACCAC
TATCTGAGCACCCAGAGCGCGCTGAGCAAAGACCCGAACGAAAAGCGTGATCACATG
GTGCTGCTGGAGTTCGTTACCGCGGCGGGTATCACCCTGGGTATGGACGAGCTGTACA
AA

4. The sequences of P2Ca-EGFP:

(represent the P2Ca sequence; represent the EGFP sequence.)

AATCTGCAGCAGCAGCGCCAGCACGGCAAGAACGGCAACCAGCTCGAGTACAGTGGG
GGTGGAGGCTCTATGGTTAGCAAGGGCGAGGAACTGTTCACCGGCGTGGTTCCGATTC
TGGTGGAACTGGACGGTGATGTTAACGGCCACAAATTTAGCGTGAGCGGCGAAGGCG
AGGGCGATGCGACCTATGGTAAACTGACCCTGAAGTTCATCTGCACCACCGGTAAACT
GCCGGTGCCGTGGCCGACCCTGGTTACCACCCTGACCTACGGTGTGCAATGCTTCAGC
CGTTATCCGGACCACATGAAACAGCACGATTTCTTTAAGAGCGCGATGCCGGAAGGTT
ACGTTCAAGAGCGTACCATTTTCTTTAAAGACGATGGCAACTATAAGACCCGTGCGGA
AGTTAAATTCGAGGGTGACACCCTGGTGAACCGTATCGAACTGAAAGGCATTGACTTT
AAAGAGGACGGTAACATCCTGGGCCACAAACTGGAATACAACTATAACAGCCACAAC
GTTTACATCATGGCGGATAAACAAAAGAACGGCATTAAAGTGAACTTTAAGATCCGTC
ACAACATTGAGGACGGTAGCGTTCAGCTGGCGGATCACTACCAGCAAAACACCCCGAT
TGGTGACGGCCCGGTTCTGCTGCCGGATAACCACTATCTGAGCACCCAGAGCGCGCTG
AGCAAAGACCCGAACGAAAAGCGTGATCACATGGTGCTGCTGGAGTTCGTTACCGCG
GCGGGTATCACCCTGGGTATGGACGAGCTGTACAAA

5. The sequences of P2Cb-EGFP:

(represent the P2Cb sequence; represent the EGFP sequence.)

GACTACCAGGATCAGAGCAACGAACAGAGGAAGAACCAGAGGCTCGAGTACAGTGG
GGGTGGAGGCTCTATGGTTAGCAAGGGCGAGGAACTGTTCACCGGCGTGGTTCCGATT
CTGGTGGAACTGGACGGTGATGTTAACGGCCACAAATTTAGCGTGAGCGGCGAAGGC
GAGGGCGATGCGACCTATGGTAAACTGACCCTGAAGTTCATCTGCACCACCGGTAAAC
TGCCGGTGCCGTGGCCGACCCTGGTTACCACCCTGACCTACGGTGTGCAATGCTTCAG
CCGTTATCCGGACCACATGAAACAGCACGATTTCTTTAAGAGCGCGATGCCGGAAGGT
TACGTTCAAGAGCGTACCATTTTCTTTAAAGACGATGGCAACTATAAGACCCGTGCGGA
AGTTAAATTCGAGGGTGACACCCTGGTGAACCGTATCGAACTGAAAGGCATTGACTTT
AAAGAGGACGGTAACATCCTGGGCCACAAACTGGAATACAACTATAACAGCCACAAC
GTTTACATCATGGCGGATAAACAAAAGAACGGCATTAAAGTGAACTTTAAGATCCGTC

ACAACATTGAGGACGGTAGCGTTCAGCTGGCGGATCACTACCAGCAAAACACCCCGAT
TGGTGACGGCCCGGTTCTGCTGCCGGATAACCACTATCTGAGCACCCAGAGCGCGCTG
AGCAAAGACCCGAACGAAAAGCGTGATCACATGGTGCTGCTGGAGTTCGTTACCGCG
GCGGGTATCACCCTGGGTATGGACGAGCTGTACAAA

6. The sequences of P2Cc-EGFP:

(represent the P2Cc sequence; represent the EGFP sequence.)

AGGACCAGCAGCGAGGAGGACTACAGCGAGGAGGTTAAGAACCTCGAGTACAGTGG
GGGTGGAGGCTCTATGGTTAGCAAGGGCGAGGAACTGTTCACCGGCGTGGTTCCGATT
CTGGTGGAACTGGACGGTGATGTTAACGGCCACAAATTTAGCGTGAGCGGCGAAGGC
GAGGGCGATGCGACCTATGGTAAACTGACCCTGAAGTTCATCTGCACCACCGGTAAAC
TGCCGGTGCCGTGGCCGACCCTGGTTACCACCCTGACCTACGGTGTGCAATGCTTCAG
CCGTTATCCGGACCACATGAAACAGCACGATTTCTTTAAGAGCGCGATGCCGGAAGGT
TACGTTCAAGAGCGTACCATTTTCTTTAAAGACGATGGCAACTATAAGACCCGTGCGGA
AGTTAAATTCGAGGGTGACACCCTGGTGAACCGTATCGAACTGAAAGGCATTGACTTT
AAAGAGGACGGTAACATCCTGGGCCACAAACTGGAATACAACTATAACAGCCACAAC
GTTTACATCATGGCGGATAAACAAAAGAACGGCATTAAAGTGAACTTTAAGATCCGTC
ACAACATTGAGGACGGTAGCGTTCAGCTGGCGGATCACTACCAGCAAAACACCCCGAT
TGGTGACGGCCCGGTTCTGCTGCCGGATAACCACTATCTGAGCACCCAGAGCGCGCTG
AGCAAAGACCCGAACGAAAAGCGTGATCACATGGTGCTGCTGGAGTTCGTTACCGCG
GCGGGTATCACCCTGGGTATGGACGAGCTGTACAAA

Supplementary Materials S2

Cas9 Fusion Protein Expression and Western Blot Validation

SDS-PAGE analysis revealed successful expression of Cas9 fusion proteins following IPTG induction. Results showed that overnight IPTG induction induced protein expression at molecular weight of 140 kDa (Cas9-P2C-His) and 163 kDa (Cas9-P2C-EGFP-His), consistent with predicted sizes of the constructs. In addition, Cas9-EGFP-His fusion proteins containing the truncated P2Ca, P2Cb, and P2Cc peptides were also successfully expressed, with major bands detected at approximately 160 kDa (Supplementary Figure S1 A). Western blot analysis further confirmed the identity of all five Cas9 fusion proteins, supporting their successful expression and integrity (Supplementary Figure S1B).

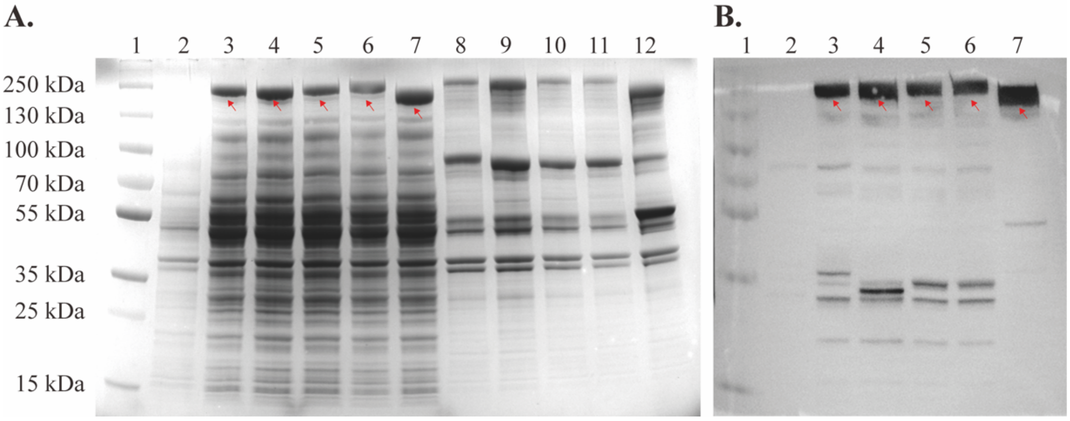

Supplementary Figure S1. Cas9 fusion protein expression and Western blot identification. (**A**). SDS-PAGE analysis of induced Cas9 fusion proteins. Lane 1: protein marker; lane 2: uninduced bacterial protein expression; lane 3-7: soluble fractions of induced P2Ca-Cas9-EGFP-His, P2Cb-Cas9-EGFP-His, P2Cc-Cas9-EGFP-His, P2C-Cas9-EGFP-His, P2C-Cas9-His; lane 8-12: corresponding insoluble fractions of the same induced proteins. (**B**). Western blot analysis using anti-Cas9 monoclonal antibody. Lane 1: protein marker; lane 2: Uninduced bacterial protein; lane 3-7: soluble fractions of induced P2Ca-Cas9-EGFP-His, P2Cb-Cas9-EGFP-His, P2Cc-Cas9-EGFP-His, P2C-Cas9-EGFP-His, P2C-Cas9-His, respectively.

Supplementary Materials S3

Cas9 Fusion Protein Purification and Dialysis

SDS-PAGE analysis revealed substantial contaminating proteins in the *E. coli* supernatant, resulting in low purity of the Cas9-P2C fusion protein prior to purification. Following the His-tag affinity purification, a marked reduction in contaminants was observed across all five Cas9 fusion protein preparations, indicating effective enrichment of the target proteins (Supplementary Figure S2A-E). Because imidazole is introduced during His-tag elution, the purified proteins were subsequently subjected to dialysis. After 24 h of dialysis, the imidazole levels decreased significantly, consistent with the requirements for subsequent experimental use.

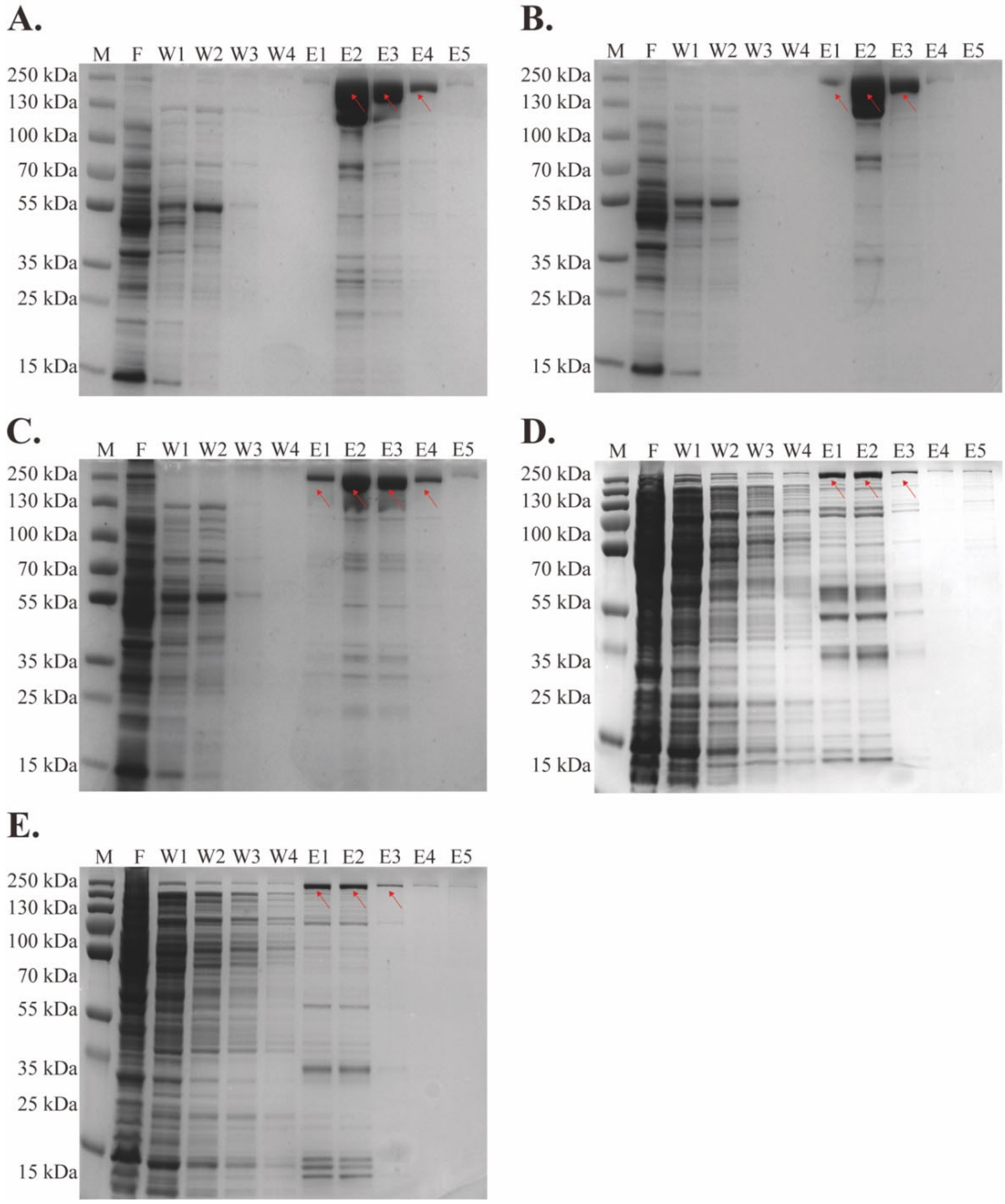

Supplementary Figure S2. SDS-PAGE analysis of Cas9 fusion protein purification. (**A**) Cas9-P2Ca-EGFP-His purification. (**B**) Cas9-P2Cb-EGFP-His purification. (**C**) Cas9-P2Cc-EGFP-His purification. (**D**) Cas9-P2C-EGFP-His purification. (**E**) Cas9-P2C-His purification. (M: Marker; F: Flow-through fraction; W_1-W_4: Wash fractions for 1st time to 4th time; E_1-E_5: Elution fractions for 1st time to 5th.)
