# Supplementary figures and images for: Methodological Evaluation of a P2C-Based ReMOT CRISPR/Cas9 System in Aedes aegypti
Source: Insects. 2026 Apr 24;17(5):451. doi: 10.3390/insects17050451 (PMC13207695; doi:10.3390/insects17050451)

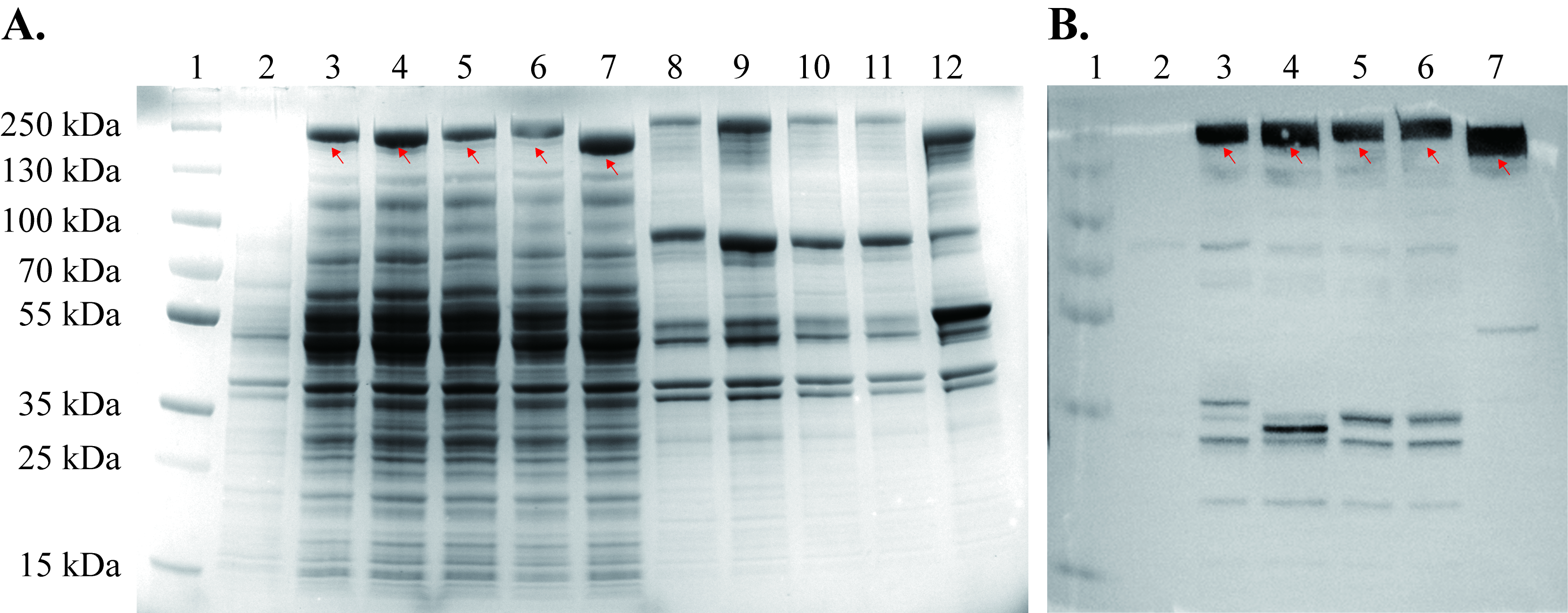

Supplement: Supplementary file 1 [file insects-17-00451-s001.zip › Supplementary Figure S1.tif]
